# Radiographic Characterization of Rib Structural Deformity Following Surgical Patent Ductus Arteriosus Clipping in Preterm Infants

**DOI:** 10.7759/cureus.88021

**Published:** 2025-07-15

**Authors:** Naoto Nishizaki, Michimasa Suzuki, Nao Kikuchi, Daishi Hirano, Reina Mayumi, Keisuke Nakanishi, Ken Takahashi, Hiromichi Shoji

**Affiliations:** 1 Pediatrics, Juntendo University Urayasu Hospital, Chiba, JPN; 2 Radiology, Juntendo University Urayasu Hospital, Chiba, JPN; 3 Pediatrics, The Jikei University School of Medicine, Tokyo, JPN; 4 Cardiovascular Surgery, Juntendo University Faculty of Medicine, Tokyo, JPN; 5 Pediatrics, Juntendo University Faculty of Medicine, Tokyo, JPN

**Keywords:** bone deformity, chest radiography, heart surgery, patent ductus arteriosus, preterm infants, preterm neonates, rib deformity, surgical clipping, surgical ligation

## Abstract

Background: The effect of patent ductus arteriosus (PDA) ligation through clipping on rib structural integrity in preterm infants is not yet fully elucidated. This study aimed to analyze radiographically evident rib deformity in preterm infants following PDA ligation and identify factors associated with these modifications.

Methods: This retrospective observational study enrolled preterm infants (gestational age <37 weeks) who underwent PDA ligation through left posterolateral thoracotomy between January 2014 and December 2022. Two radiologists, blinded to the clinical data, independently evaluated serial plain chest radiographs obtained before and after the surgical intervention.

Results: Of the 46 preterm infants with PDA requiring surgical ligation, 28 met the inclusion criteria. Six patients exhibited radiographic rib deformity following the procedure (rib alteration cohort), whereas 22 exhibited no such changes (control cohort). The structural modifications predominantly affected the left third through sixth ribs. Although no significant intergroup differences were observed in gestational age, birthweight, sex, antenatal corticosteroid administration, cumulative nonsteroidal anti-inflammatory drug (NSAID) doses, chronological age at surgery, operative duration, or incidence of postoperative complications, the rib deformity cohort demonstrated significantly lower utilization of preoperative NSAIDs (P = 0.04) and significantly higher prevalence of retinopathy of prematurity requiring intervention at discharge (P < 0.01). In addition, the corrected gestational age at surgical intervention tended to be younger in the cohort with rib deformities than in controls (P = 0.05).

Conclusion: Rib structural deformity represents a common phenomenon in preterm infants who did not receive NSAIDs before PDA ligation and those with retinopathy of prematurity. Furthermore, these modifications tended to occur in infants with a younger corrected gestational age at the time of surgical intervention.

## Introduction

Patent ductus arteriosus (PDA) is a significant cardiovascular challenge in preterm infant care, affecting up to 70% of infants born before 28 weeks of gestation [1]. Hemodynamically significant left-to-right shunting through the persistent ductus arteriosus increases mortality risks and predisposes patients to severe morbidities, such as intraventricular hemorrhage (IVH), necrotizing enterocolitis, chronic lung disease, and retinopathy of prematurity (ROP) [1-3]. The therapeutic approach to PDA management follows a hierarchical algorithm, initially comprised of pharmacological intervention with nonsteroidal anti-inflammatory drugs (NSAIDs) or acetaminophen. However, approximately 20%-40% of cases exhibit refractoriness to medical therapy, necessitating surgical ligation through clipping [4]. Although surgical management achieves definitive ductal closure, the procedure involves manipulation of the thoracic cage near developing osseous structures, potentially affecting the surrounding anatomical elements. Surgical PDA clipping is at risk of immediate complications, such as bleeding, chylothorax, pneumothorax, and inadvertent occlusion of the left main bronchus, left pulmonary artery, or aorta [5-8]. Furthermore, postoperative complications include acute left ventricular dysfunction, left vocal cord paresis, upper airway obstruction, and respiratory failure at the time of endotracheal extubation; however, the effects of thoracotomy on the thoracic cage of premature infants are still unclear [9-11]. The identification of the clinical parameters associated with postsurgical rib structural modifications carries substantial clinical relevance. Elucidating these associations could provide information for preoperative risk stratification, modify surgical techniques, and guide postoperative surveillance protocols.

This study aimed to quantify and characterize radiographically evident rib deformity following surgical PDA ligation in preterm infants, identify associated clinical and perioperative factors, and assess their temporal progression during the immediate postoperative period.

## Materials and methods

Study population

A retrospective observational study was conducted in the neonatal intensive care unit of Juntendo University Urayasu Hospital, a tertiary hospital in Chiba Prefecture in Japan, between January 2014 and December 2022. The study cohort comprised preterm infants (gestational age <37 weeks) who underwent PDA ligation through clipping. Infants who had chromosome anomalies or genetic disorders, concomitant cardiac malformations, and additional surgical interventions during the study period, died before hospital discharge, or had insufficient medical documentation were excluded. For comparative analysis, patients were stratified into two cohorts based on the presence or absence of radiographically confirmed rib deformity following PDA ligation.

Data collection

Comprehensive clinical data were extracted from electronic medical records, including gestational age, birthweight, gender, maternal factors, and perinatal characteristics such as steroid administration and Apgar scores. The focus was on initial therapeutic interventions, including exogenous surfactant administration, inhaled nitric oxide therapy, and mechanical ventilatory support requirements. PDA-specific parameters were documented in detail, encompassing NSAIDs administration patterns, surgical timing, and operative characteristics. Postoperative complications and clinical outcomes were systematically recorded, including the development of chronic lung disease (defined as oxygen dependence for ≥28 days) [12], IVH (Papile’s grade ≥1) [13], and ROP requiring photocoagulation [14]. Perioperative mortality was defined as death occurring within seven days after the surgical procedure. Laboratory investigations focused on bone metabolism markers, including serum calcium (Ca; reference range, 9.0-11.0 mg/dL), serum phosphorus (P; reference range, 5.0-7.7 mg/dL), and alkaline phosphatase (ALP; reference range, 186-564 U/L). These biochemical parameters were assessed preoperatively on the day of surgery, postoperatively within 24 hours, and at the time of rib deformity detection within a three-day interval in accordance with a standardized blood test protocol within the facility.

Pharmacotherapy for PDA closure

The initiation of pharmacotherapy for PDA was based on a combination of clinical and echocardiographic criteria. Clinical indicators included severe symptoms such as pulmonary hemorrhage or severe respiratory insufficiency, concomitant with signs of systemic hypoperfusion, which could be observed as acute kidney injury or refractory hypotension. Echocardiographic parameters warranting therapeutic intervention consisted of duct diameter >1.5 mm or left atrium to aortic root ratio >1.5, initially assessed within six hours postnatally and subsequently monitored at 12-hour intervals until ductal closure was documented. The pharmacotherapeutic protocol involved the intravenous administration of indomethacin or ibuprofen, allowing for up to three therapeutic courses with a maximum cumulative dose of nine administrations per patient [15]. In this study, PDA was not treated with acetaminophen because it was not approved by the Japanese Pharmaceutical Affairs Law during the period of this study. This pharmacological regimen continued in the absence of contraindications, such as IVH, gastrointestinal complications, or significant laboratory derangements. Surgical ligation through clipping was reserved for cases exhibiting severe symptoms that contraindicated NSAIDs or those that demonstrated refractoriness to pharmacological management.

Surgical technique for PDA closure

All ligation procedures of the PDA were performed utilizing a bedside approach by the cardiovascular surgeon accompanied by a surgical assistant. The standardized operative technique employed a left mid-axillary minithoracotomy along the fourth intercostal space, culminating in mechanical clip ligation of the ductus arteriosus. To facilitate subsequent analysis of the operative time as a potential determinant of clinical outcomes, the duration of the procedure was meticulously documented for each intervention.

Chest radiography and evaluation

Our radiographic imaging protocol strictly adhered to the Japan Diagnostic Reference Levels [16], utilizing a portable imaging device (MobileArt Evolution; Shimadzu Corporation, Kyoto, Japan) with radiation exposure doses maintained <0.2 mGy per examination. To ensure the analysis of standardized evaluation parameters, digital images were processed using the SYNAPSE system (Fujifilm Medical, Tokyo, Japan) and displayed on a Radiforce RX850 monitor (EIZO Corporation, Ishikawa, Japan). Serial chest radiographs (frontal anteroposterior view) were taken from the preoperative period until hospital discharge, which were independently evaluated by two radiologists who were blinded to the clinical data. Comparison of post-ligation radiographs with preoperative baseline imaging revealed that rib deformities were specifically characterized as structural modifications, including cortical narrowing, osseous fusion, or complete absence.

Statistical analysis

The study participants were stratified according to the radiographic findings into cohorts with or without rib deformities. Summary statistics are presented as median values (interquartile range) for continuous variables and as frequency (percentage) for categorical variables. We performed the chi-square test or Fisher’s exact test (as appropriate) for categorical variables, independent sample t-tests for normally distributed continuous variables, and Wilcoxon-Mann-Whitney U tests for non-normally distributed continuous variables. All statistical analyses were performed using Statcel 4 software (OMS Publishing Inc., Saitama, Japan). P < 0.05 was considered significant.

Ethics approval and consent

This study was approved by the Institutional Review Board of the Juntendo University School of Medicine (Approval no. E22-0427) and was conducted in accordance with the tenets of the 1964 Declaration of Helsinki and its later amendments or comparable ethical standards. The requirement for written informed consent was waived owing to the study’s retrospective design and the opt-out approach.

## Results

**Figure 1:**
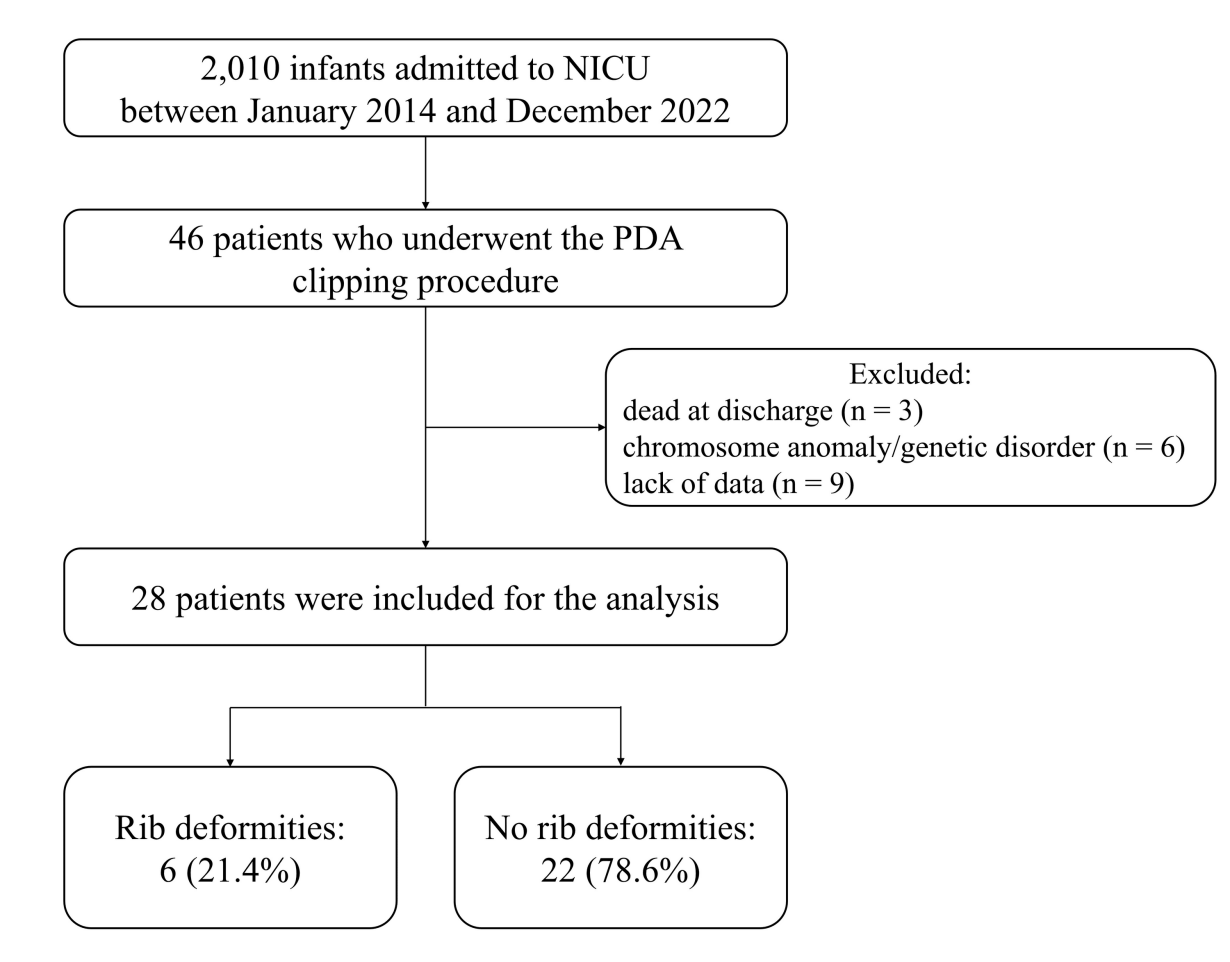
Flow diagram of participant selection and study cohort derivation. NICU, neonatal intensive care unit; PDA, patent ductus arteriosus.

Figure 1 shows the flowchart of study enrollment. The cohort comprised 46 preterm infants who underwent PDA clipping during the study period. Of these infants, 18 were excluded based on the exclusion criteria. Finally, 28 patients were included in the analysis and were divided into groups with (n = 6) and without (n = 22) rib deformities.

Table 1 summarizes the demographic characteristics of the study population with rib deformities versus those without rib deformities after PDA clipping. The prevalence of pharmacotherapy was significantly lower in the group with rib deformities taking NSAIDs preoperatively than in the group without rib deformities (66.7 versus 100%, P = 0.04). Two of the six patients with rib deformities did not receive NSAIDs before PDA clipping because of anuria in one case and paralytic ileus with biliary gastric contents in the other. For PDA clipping, no significant differences were observed in patient age, weight, or operation time. However, although no significant difference was noted, the group with rib deformities tended to be younger than the group without rib deformities in terms of the number of corrected gestational age at the time of surgery (25.8 versus 27.8 weeks, P = 0.05). No hospital mortality was recorded, and the incidence of ROP at discharge was significantly higher in the group with rib deformities than in the group without such deformities (83.3 versus 18.2%, P < 0.01).

**Table 1:** Demographic characteristics of the study population (N = 28). SGA, small for gestational age; PDA, patent ductus arteriosus; NO, nitric oxide; NSAIDs, nonsteroidal anti-inflammatory drugs; CLD, chronic lung disease; IVH, intraventricular hemorrhage; ROP, retinopathy of prematurity; N/A, not applicable. Categorical variables: Data are displayed by count (n) and percentage (%). The chi-square test or Fisher’s exact test (as appropriate) was conducted to compare percentage distributions between groups with rib deformities versus those without rib deformities after PDA clipping. Continuous variables (normally distributed): Data are presented by median values (interquartile range). Independent samples Mann–Whitney U tests were conducted to compare the mean scores between groups with rib deformities and those without rib deformities after PDA clipping. Continuous variables (not normally distributed): Data are displayed by median values (interquartile range). Wilcoxon Mann–Whitney U tests were performed to compare the median values between groups with rib deformities versus those without rib deformities after PDA clipping. The distribution of continuous variables was compared using Wilcoxon Mann–Whitney U tests. A p-value of <0.05 was considered statistically significant.

Characteristics	All N = 28 (100%)	Rib deformities N = 6 (21.4%)	No rib deformities N = 22 (78.6%)	p-value
					Gestational age at birth (weeks) (median values [interquartile range])	25 (24-28)	24.5 (22.8-25)	26 (24-28)	0.07
Birth weight (gram) (median values [interquartile range])	731 (563-992)	699 (504-810)	760 (564-1,055)	0.37
Male, n (%)	12 (43)	5 (83.3)	11 (50)	0.16
Maternal age (year) (median values [interquartile range])	32.5 (29-36)	34.5 (28.5-37.3)	31.5 (29-36.3)	0.59
Caesarean delivery, n (%)	26 (93)	5 (83.3)	21 (95.5)	0.39
Singleton, n (%)	25 (89)	6 (100)	19 (86.4)	0.47
SGA, n (%)	2 (7)	0 (0)	2 (9.1)	0.61
Prenatal steroids administration, n (%)	18 (64)	2 (33.3)	16 (72.7)	0.97
Apgar score at 5 min (median values [interquartile range])	7 (6-8)	7 (5-7)	7 (6-8)	0.21
Surfactant replacement before PDAclipping, n (%)	25 (89)	6 (100)	19 (86.4)	0.47
NO inhalation before PDA clipping, n (%)	7 (25)	1 (16.7)	6 (27.3)	0.52
Mechanical ventilation required before PDA clipping, n (%)	28 (100)	6 (100)	22 (100)	0.63
Continuous positive pressure ventilation, n (%)	13 (46)	3 (50)	10 (45)	0.60
High frequency oscillatory ventilation, n (%)	15 (54)	3 (50)	12 (55)	0.60
NSAIDs administration before PDA clipping, n (%)	26 (92.9)	4 (66.7)	22 (100)	0.04
Age at first administration of NSAIDs (day of life) (median values [interquartile range])	1 (0-1)	1 (0-1.3)	1 (0-1.3)	0.46
Age at last administration of NSAIDs (day of life) (median values [interquartile range])	4.5 (3.3-7)	4 (3.3-7.3)	5 (4-7.3)	0.61
Type of NSAIDs				
Indomethacin, n (%)	10 (38.5)	2 (33.3)	8 (36.4)	0.50
Ibuprofen, n (%)	16 (61.5)	2 (33.3)	14 (63.6)	0.50
Frequency of NSAIDs administration (median values [interquartile range])	4 (3-6)	3.5 (2.75-6)	4 (3-6)	0.64
Age at PDA clipping (days) (median values [interquartile range])	12.5 (8-19.5)	15 (5.8-24.5)	11 (8-19)	0.73
Age at PDA clipping (corrected gestational age) (median values [interquartile range])	27 (25-29)	26 (24.8-26.5)	27 (25.8-29.3)	0.05
Body weight at PDA clipping (gram) (median values [interquartile range])	713.5 (566-1,020)	697.5 (559-769)	759.5 (569-1,084)	0.31
Operation time (min)	30.5 (29-37)	31.5 (23.8-38)	30.5 (29-36)	0.57
Postoperative complications				
Pneumothorax, n (%)	2 (7.1)	1 (16.7)	1(4.5)	0.39
Left vocal cord paresis, n (%)	3 (10.7)	0 (0)	3 (13.6)	0.47
Complications at discharge				
CLD, n (%)	4 (14.3)	1 (16.7)	3 (13.6)	0.81
IVH, n (%)	5 (17.9)	2 (33.3)	3 (13.6)	0.95
ROP, n (%)	9 (32.1)	5 (83.3)	4 (18.2)	<0.01
Tracheotomy, n (%)	0 (0)	0 (0)	0 (0)	N/A
Hospital mortality, n (%)	0 (0)	0 (0)	0 (0)	N/A
Duration of hospitalization (days) (median values [interquartile range])	128 (99-179)	160.5 (117.8-218.5)	118.5 (94-176.3)	0.17

Table 2 shows the background and laboratory data of six cases with rib deformities on chest radiographs. The median age at the time of PDA clipping was 15 days, and the rib deformities were detected for the first time at a median of 57 days postoperatively. The six patients with rib deformities had no abnormalities in their serum concentrations of Ca, P, and ALP in the preoperative period, postoperative period, and period when the rib deformities were recognized.

**Table 2:** Background and laboratory data of six cases with rib deformities on chest radiograph. Ca, calcium; P, phosphorus; ALP, alkaline phosphatase

No. of case	Gestational age at birth (weeks)	Birth weight (grams)	Gender		Preoperative serum concentration				Postoperative serum concentration				Serum concentration at the time of recognition of rib deformities		
					Ca (mg/dL)	P (mg/dL)	ALP (U/L)		Ca (mg/dL)	P (mg/dL)	ALP (U/L)		Ca (mg/dL)	P (mg/dL)	ALP (U/L)
1	22	505	boy		9.1	6.2	408		4.6	7.2	364		9.8	5.9	475
2	25	683	boy		10.1	4.3	228		3.9	10.2	324		8.8	4.5	991
3	24	777	boy		9.3	2.6	142		5.7	11.1	137		9.1	4.3	445
4	25	907	boy		6.9	3.5	277		4.3	6.9	382		8.5	5.3	441
5	23	499	boy		8.6	5.9	426		3.3	7.6	321		8.5	4.8	868
6	25	715	girl		11.1	2.2	250		1.7	9.2	384		8.5	4.9	461

**Figure 2:**
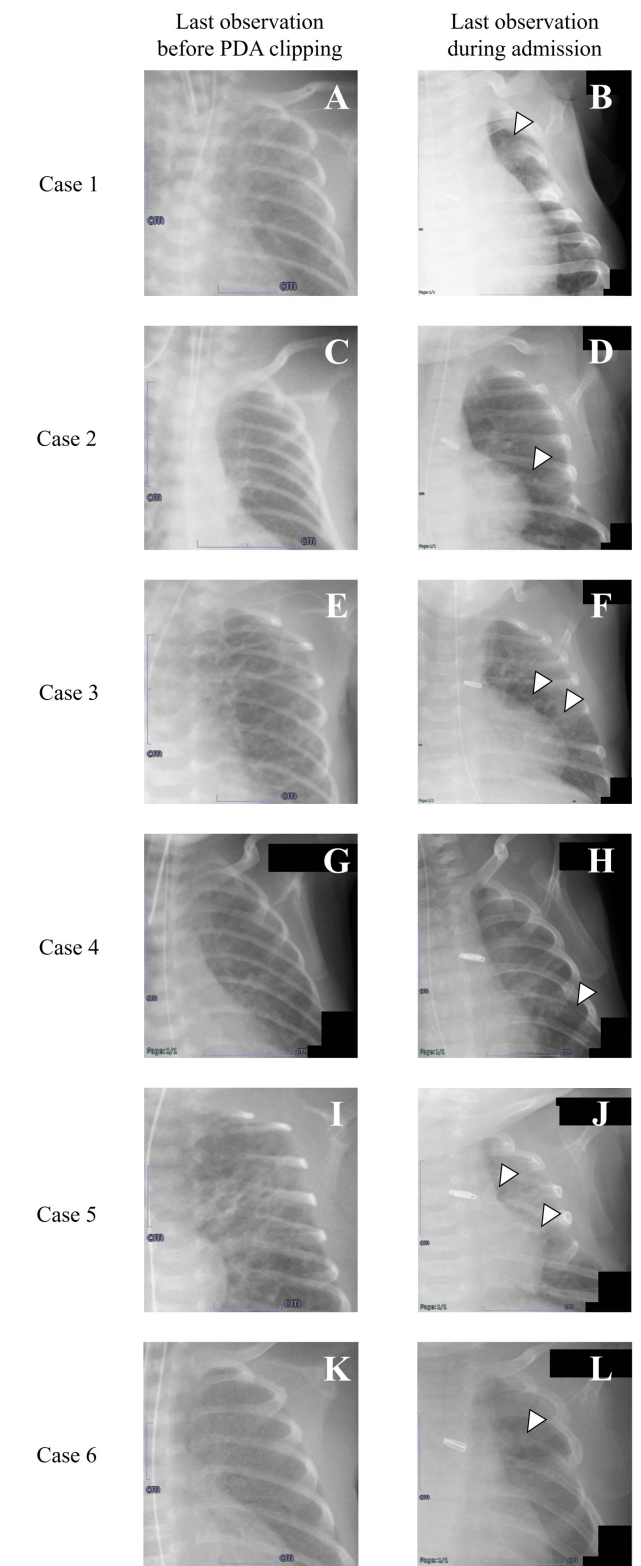
Serial plain chest radiographs demonstrating the temporal evolution of structural rib deformities in six preterm infants during hospitalization. White arrowheads indicate sites of radiographically evident rib modifications. Case 1 (panels A and B) shows disappearance of the third rib; Case 2 (panels C and D) shows narrowing of the sixth rib; Case 3 (panels E and F) shows narrowing of the fifth rib and disappearance of the sixth rib; Case 4 (panels G and H) shows disappearance of the sixth rib; Case 5 (panels I and J) shows disappearance of the fourth rib and narrowing of the fifth rib; Case 6 (panels K and L) shows disappearance of the fourth rib. PDA, patent ductus arteriosus.

Figure 2 presents a time series of the images of the six patients with eight rib deformities after PDA clipping. The six cases with rib changes were consistent with the opinions of two radiologists. In cases 3 and 5, two rib deformities were observed in each case. In all cases, the changes occurred in the left third to sixth ribs. In these patients, the types of rib deformity in were narrowing (n = 3) and disappearance (n = 5). None of the deformity types had changed at the last observation during hospitalization.

## Discussion

This study presents three principal findings regarding rib deformities following surgical PDA ligation through clipping in preterm infants. First, significant structural modifications of the ribs occurred in 21.4% of the cases, predominantly affecting the left third to sixth ribs. Second, this deformity demonstrated significant associations with the absence of preoperative NSAIDs administration and the presence of ROP. Third, the timing of surgical intervention, specifically younger corrected gestational age, exhibited a trend toward increased risk of rib deformity.

This study identified two distinct morphological patterns in patients who underwent PDA clipping: cortical narrowing and complete radiographic disappearance. A plausible mechanism for these structural modifications involves the intraoperative insertion of surgical retractors between the ribs to improve the visualization of the PDA. In preterm infants, whose skeletal structures demonstrate increased plasticity, placement of retractors likely induces varying degrees of trauma to the adjacent costal elements [17]. Indeed, several studies have suggested that osseous deformation is indirectly mediated by factors such as regional perfusion deformity and pressure distribution dynamics [18-20]. Furthermore, the standard surgical technique involves closing the deeper fascial and muscular layers using absorbable sutures after ligation. It is postulated that suture placement under tension, although necessary to prevent tissue dehiscence, may be associated with localized tissue injury. These factors may induce osseous malalignment and disrupt normal osteogenic processes, particularly in the preterm population, thereby contributing to postoperative rib deformity. In addition, when the latissimus dorsi and/or serratus anterior muscles are cut during a posterolateral thoracotomy, local muscle atrophy and deformation of the thorax and/or spine may occur after surgery [11]. However, secondary bone alterations associated with muscular atrophy following the incision tend to occur after infancy; therefore, the results of this study do not fully explain the phenomenon.

The present study demonstrated the involvement of the third and sixth ribs. Although rib abnormalities on plain radiography in infants can suggest traumatic etiology [21], the median Apgar score at five minutes for the six patients with rib deformity was 7 points, and no documented resuscitative interventions required thoracic compressions. In addition, serum Ca, P, and ALP concentrations related to bone metabolism remained predominantly within the reference ranges throughout the perioperative period. Although the precise mechanism underlying ribs beyond the fourth and fifth intercostal spaces remains unclear, potential explanations include inadvertent application of pressure to the thoracic wall surrounding the operative field or unintentional trauma from medical instrumentation when the patient is obscured by sterile draping during the procedure. These circumstances may result in pressure-induced injury or inhibition of the normal development of the costal elements and surrounding tissues, resulting in osseous deformity. As these situations may be partially mitigated through a modified technique, a novel finding of the present investigation is that rib deformity following PDA clipping in preterm infants may manifest in a broader anatomical distribution than previously documented.

An intriguing finding of this study is the higher incidence of rib structural deformities among patients who did not receive preoperative NSAIDs. This appears paradoxical considering previous studies reporting that NSAIDs inhibit bone remodeling and fusion [22-24]. However, NSAIDs are considered unlikely to contribute significantly to rib deformities. Furthermore, the association observed between NSAID administration and rib deformities in this study is likely to reflect random variation rather than a true causal relationship. The precise biological mechanism underlying this association remains unclear and warrants further investigations through larger prospective studies examining the complex interplay between NSAIDs, surgical intervention, and developing skeletal structures in preterm infants.

In this study, rib deformities showed a significant association with the presence of ROP. There was no difference in the frequency of use of continuous positive pressure ventilation and high-frequency oscillatory ventilation between the two groups. Infants with high lung compliance are more likely to be exposed to negative pressure in the window area during thoracotomy, which may increase the likelihood of subsequent rib deformities. Such infants are also more prone to unstable oxygen saturation, which may increase the risk of ROP development.

The long-term musculoskeletal sequelae following surgical PDA ligation in preterm infants remain incompletely characterized. Seghaye et al. reported thoracic abnormalities on plain radiographs following surgical PDA ligation in infants (median chronological age six years and one month) [25]. According to their findings, 20 of 27 children demonstrated rib anomalies at the level of the left-sided thoracotomy, even in one patient diagnosed with thoracic scoliosis. Similarly, Jaureguizar et al. identified rib abnormalities on chest radiographs obtained during a 16-year postoperative period following thoracotomy for tracheoesophageal fistula repair [18]. In the present study, six of 28 (21.4%) patients developed rib deformities during the relatively brief observational period following PDA clipping. Despite the potential association of rib deformities with long-term pulmonary function and musculoskeletal complications, late-onset sequelae following PDA clipping have not been systematically investigated. Therefore, prospective longitudinal studies are warranted to elucidate the long-term evolution of rib deformities following surgical PDA ligation in preterm infants.

This study has several limitations that warrant our consideration. First, the single-center retrospective design and relatively limited sample size constrain the generalizability of our findings. Second, due to the small sample size of the two groups, multivariate analysis was deemed inappropriate, and statistical analysis to confirm the causal relationship between each parameter and rib deformity could not be performed. Third, the observational period was limited to the hospitalization period, leaving questions about long-term outcomes unanswered. Finally, the inability to standardize the surgical technique across all cases may have introduced uncontrolled variability in our results. Future research should include prospective multicenter studies with standardized surgical protocols and extended follow-up periods. Investigation of potential protective interventions, particularly in high-risk patients, is warranted. In addition, a detailed analysis of the mechanical forces applied during surgery and their relationship to structural changes could inform modifications in the surgical technique.

## Conclusions

This study investigation demonstrates that a significant proportion of preterm infants develop radiographically evident rib deformities following surgical PDA ligation. These structural modifications warrant systematic longitudinal surveillance to elucidate their natural history and potential long-term sequelae. More prospective studies are essential to elucidate the clinical significance of these findings and inform evidence-based modifications to surgical approaches in this vulnerable population.
